# Delayed medical care and underlying health in the United States during the COVID-19 pandemic: A cross-sectional study

**DOI:** 10.1016/j.pmedr.2022.101882

**Published:** 2022-07-05

**Authors:** Autumn H. Gertz, Catherine C. Pollack, Marinanicole D. Schultheiss, John S. Brownstein

**Affiliations:** a Computational Epidemiology Lab, Boston Children’s Hospital, Boston, MA, USA; b Department of Biomedical Data Science, Geisel School of Medicine at Dartmouth, Hanover, NH, USA; c Department of Epidemiology, Geisel School of Medicine at Dartmouth, Hanover, NH, USA; d Harvard Medical School, Harvard University, Boston, MA, USA

**Keywords:** Delayed medical care, COVID-19, Underlying health conditions, Health outcomes

## Abstract

This study assesses the association between underlying health conditions and delaying medical care during the COVID-19 pandemic. An online cross-sectional survey administered by OutbreaksNearMe.org on Momentive.ai collected self-reported data from April 27 to June 2, 2020 and May 10 to June 13, 2021. We used weighted multivariable logistic regressions to assess the association between delaying care and self-reported health status, adjusting for demographics. Of 312,661 total responses (99.6% completion rate), 17.1% reported delayed medical care. Compared to good health, those with poor health were more likely to delay care (AOR = 2.62, 95% CI [2.47, 2.78]). Individuals with any underlying condition (AOR = 1.62, 95% CI [1.58, 1.65]) and each of the conditions were more likely to delay care. Differences in delaying care were observed across region, year, and demographics. Our finding is that those at higher risk of severe COVID-19 were more likely to delay medical care in 2020 and 2021, which could exacerbate existing health conditions and existing disparities.

## Highlights

• An online cross-sectional survey administered twice during the COVID-19 pandemic.
• Those with poor health were more likely to delay care than those with good health.
• Those with underlying conditions were more likely to delay medical care.
• Across underlying conditions, odds of delaying care increased between 2020 and 2021.

## 1 Introduction

The COVID-19 pandemic affected access and receival of medical care with various layers of healthcare systems impacted (Czeisler, 2020, Jiang et al., 2020). Early in the pandemic, visits to medical services decreased (Hartnett, 2020, Epic Research, 2022, The Impact of the COVID-19 Pandemic on Outpatient Visits: A Rebound Emerges, Santoli, 2020, acep-mc-covid19-april-poll-analysis.pdf), and some medical providers delayed or rescheduled appointments to decrease exposure in the office and prepare for an expected increase of cases (COVID-19 significantly impacts health services for noncommunicable diseases, 2022, Masroor, 2020). By June 30, 2020, 41% of adults in the United States (U.S.) reported delaying or avoiding medical care due to COVID-19 concerns (Czeisler, 2020). Delays in medical care may increase morbidity and mortality risk among those with underlying, preventable, and treatable medical conditions (Czeisler, 2020, Masroor, 2020, CDC, 2022, Blay et al., 2021, Tapper and Asrani, 2020). Individuals with pre-existing conditions associated with severe COVID-19 may be especially susceptible (Czeisler, 2020, Masroor, 2020, CDC, 2022, Blay et al., 2021, Tapper and Asrani, 2020, CDC, 2020).

Furthermore, the pandemic exacerbated existing health disparities across sociodemographic groups. Communities already experiencing profound impacts from COVID-19, such as Black, Indigenous and People of Color or individuals of lower socioeconomic status, could be particularly affected. As variants of concern continue to emerge, it is imperative to explore the impact that delaying medical care could have on vulnerable populations.

Delayed medical care is another axis that could lead to long-term variations in health outcomes and have lasting implications on individual and public health. Previous work evaluated delayed care during 2020, but there has yet to be a comparative analysis across multiple time periods within the pandemic. Therefore, we aim to assess the association between delaying medical care during two time points in the pandemic and underlying health, demographic, and regional factors.

## 2 Methods

### 2.1 Survey population

We gathered responses from a previously validated online survey administered by OutbreaksNearMe.org on Momentive.ai (previously SurveyMonkey) (Rader et al., 2021, Chan and Brownstein, 2020, Rader, 2022). National population weights for gender, age, race, education, geography, political identification, and profession were applied from the U.S. Census Bureau’s American Community Survey to approximate the demographic composition of U.S. adults (Bureau UC, 2022). U.S. residents visiting Momentive were prompted through end-page river sampling. Among the two million daily visitors to the SurveyMonkey platform, participants are invited randomly to complete this survey following the completion of an unrelated survey. Each unique Internet Protocol address can participate once, as a proxy for household. There is no monetary incentive to complete this survey. Data were restricted to those who answered the question regarding delaying medical care and had weights available (99.6% of respondents). No additional restrictions were applied.

### 2.2 Primary Exposure: Pre-Existing conditions

The primary exposure of interest was the presence of at least one pre-existing condition. Participants were asked, “Do you have any of the following underlying conditions? (Please select all that apply.).” Underlying conditions captured included asthma, cancer (past year), chronic heart disease, chronic kidney disease, chronic lung disease, diabetes, and immunosuppressive conditions. Obesity was added in 2021. Results were collapsed into a binary variable as well as analyzed separately (see Statistical Analysis).

### 2.3 Secondary exposures: Sociodemographic and health characteristics

Individuals self-reported their demographic characteristics, including age, gender, race, income, insurance provider, education, and state of residence. To assess underlying health, a five-level Likert scale was modified to ask users, “Would you say your health in general is excellent, very good, good, fair, or poor.”.

### 2.4 Primary outcome: Delayed care

The primary outcome was delayed medical care. Participants were asked the binary question, “At any time in the last 4 weeks, did you delay getting medical care because of the coronavirus pandemic?”.

### 2.5 Study period

Data were collected between April 27 to June 2, 2020 and May 10 to June 13, 2021. Analysis was conducted for combined timeframes. Comparisons across timeframes were also performed.

### 2.6 Statistical analysis

Demographic comparisons of individuals who delayed care between years were conducted with chi-squared tests. Mixed-effects multivariable logistic regression models with a random effect for state and stratified by U.S. Census Region evaluated whether demographic groups differentially delayed care between regions. Fixed-effects multivariable logistic regressions assessed associations between delayed care and underlying conditions (in aggregate and stratified by region) as well as delayed care and overall health. All models incorporated survey weights and adjusted for age, gender, race, annual household income, insurance status, and education status. A significance threshold of 0.05 was used for all tests. Analysis was conducted in R (version 4.1.0) in the RStudio Integrated Development Environment (version 1.4.1717).

### 2.7 IRB approval

This study was approved by Boston Children’s Hospital Institutional Review Board and received a waiver of informed consent.

## 3 Results

We gathered 312,661 responses of 313,954 fielded surveys. Overall, 17.1% of respondents reported delaying medical care. This was higher in 2020 (21.7%, n = 54,934) than 2021 (7.12%, n = 6,908). Across years, the majority of respondents were female (63.5% in 2020 vs. 61.1% in 2021) and White (72.5% in 2020 vs. 64.6% in 2021). A plurality of respondents were between 45 and 64 years old (43.2% in 2020 vs. 43.1% in 2021) and received insurance from their employer (53.3% in 2020 vs. 47.7% in 2021).

There were significant differences between individuals who delayed care in 2020 compared to 2021 (Table 1). For example, 40.5% of individuals who delayed care in 2020 had an income below $50,000, compared to 51.7% in 2021. In contrast, 22.7% of individuals who reported delaying care in 2021 had an income above $100,000, compared to 29.6% in 2020. Additionally, 55.8% of individuals who delayed care in 2021 had at least one pre-existing condition compared to 37.2% who delayed care in 2020. Furthermore, 21.3% of adults 65 and older reported delaying care in 2020; this decreased to 10.7% in 2021. All demographic differences between years were statistically significant (P < 0.001).

**Table 1:** Demographic Covariates across delayed care status and year.

	2020 (N = 212,697)		2021 (N = 99,964)	
Characteristic	No Delayed Care (n = 157,763)n (Weighted %)	Delayed Care (n = 54,934)n (Weighted %)	No Delayed Care (n = 93,056)n (Weighted %)	Delayed Care (n = 6,908)n (Weighted %)
Region Northeast (Reference) Midwest South West	28,025 (17.2%)34,679 (21.1%)56,604 (38.5%)38,485 (23.1%)	10,332 (18.8%)12,021 (21.5%)18,262 (35.0%)14,340 (24.8%)	16,450 (17.4%)18,961 (21.1%)34,765 (38.3%)22,882 (23.3%)	1,226 (17.2%)1,210 (18.5%)2,247 (34.3%)2,225 (30.0%)
Pre-Existing ConditionNo (Reference) Yes	117,311 (75.4%)40,482 (24.6%)	34,776 (62.8%)20,179 (37.2%)	56,414 (63.6%)36,644 (36.4%)	3,118 (44.2%)3,790 (55.8%)
Age 13 – 17 18 – 24 25 – 44 45 – 64 (Reference) 65 and Over	3,181 (8.10%)9,213 (10.8%)44,721 (31.1%)67,940 (31.3%)32,738 (18.7%)	741 (6.44%)2,767 (9.82%)15,640 (30.4%)23,923 (32.0%)11,884 (21.3%)	1,528 (7.67%)5,166 (10.6%)24,883 (30.7%)40,386 (31.6%)21,095 (19.4%)	146 (8.81%)489 (13.1%)2,573 (41.6%)2,722 (25.8%)978 (10.7%)
GenderMale (Reference) Female Other	59,015 (48.5%)97,147 (50.3%)1,631 (1.23%)	16,177 (39.5%)38,000 (58.5%)778 (1.97%)	35,067 (47.6%)56,547 (50.6%)1,444 (1.84%)	2,109 (39.8%)4,528 (54.8%)271 (5.42%)
Race White (Reference) Black Hispanic Asian Other	112,679 (63.2%)17,028 (12.3%)13,276 (15.7%)7,422 (5.76%)7,388 (3.03%)	41,609 (68.7%)4,521 (10.3%)3,940 (12.8%)1,997 (4.72%)2,888 (3.52%)	60,374 (62.9%)13,116 (12.3%)9,249 (16.0%)4,875 (5.70%)5,444 (3.07%)	4,195 (59.7%)899 (12.1%)808 (17.5%)421 (6.12%)585 (4.56%)
Income Below 50,00050,000 – 99,999 (Reference) 100,000 and Above	45,363 (42.5%)47,696 (30.7%)55,843 (26.7%)	14,848 (40.5%)16,372 (29.8%)20,845 (29.6%)	28,990 (42.1%)26,907 (29.9%)30,579 (28.0%)	2,780 (51.7%)1,877 (25.6%)1,912 (22.7%)
Insurance SourceEmployer (Reference) Purchased oneself Medicare Medicaid or Medi-Cal TRICARE None Other No Answer	83,264 (45.1%)7,802 (10.5%)17,357 (19.7%)29,360 (7.04%)7,411 (1.73%)2,810 (8.09%)8,364 (6.50%)1,420 (1.36%)	30,116 (47.3%)5,413 (9.31%)10,890 (21.7%)2,949 (8.13%)1,096 (1.95%)2,058 (5.73%)2,129 (5.13%)301 (0.78%)	44,667 (43.5%)5,652 (10.2%)10,020 (20.1%)18,936 (8.11%)5,764 (1.97%)1,770 (7.38%)4,917 (6.74%)1,332 (1.89%)	3,021 (39.1%)810 (11.1%)1,225 (18.9%)771 (13.0%)139 (1.94%)444 (8.24%)414 (6.36%)84 (1.34%)
Education Did not finish high school High School/GED Associate’s Degree Some CollegeCollege Degree (Reference) Postgraduate Degree	4,538 (9.72%)22,145 (34.3%)13,850 (8.95%)29,229 (20.3%)47,131 (17.7%)40,900 (9.05%)	1,103 (7.87%)4,963 (26.3%)4,339 (9.32%)9,605 (21.6%)17,268 (21.7%)17,677 (13.2%)	2,739 (8.95%)14,769 (33.3%)9,035 (9.03%)17,994 (19.9%)26,440 (18.0%)22,081 (10.8%)	290 (11.9%)1,043 (29.9%)637 (9.21%)1,351 (19.5%)1,938 (17.8%)1,649 (11.7%)

### 3.1 Regional variation

In 2020, 23.3% of respondents from the Northeast delayed care, compared to 22.9% from the West, 22.0% from the Midwest and 20.2% from the South. In contrast, 7.06% of respondents from the Northeast delayed care in 2021, compared to 8.98% from the West, 6.32% from the Midwest, and 6.44% from the South. In both years, these proportions were significantly different (*P*_2020_ < 0.001; *P*_2021_ < 0.001).

There were distinct differences in delayed care across U.S. regions considering year. Unique to the South in 2020, individuals on Medicare (AOR = 1.08, 95% CI [1.01, 1.16]) and those with a postgraduate degree (AOR = 1.14, 95% CI [1.03, 1.26]) had higher odds of delaying care than those receiving insurance from their employer and those with a college degree, respectively. Distinct to the West in 2020, individuals above the age of 65 had a lower odds of delaying care (AOR = 0.88, 95% CI [0.80, 0.96]) compared to individuals between 45 and 64. Teenagers in the Midwest had a higher odds of delaying care (AOR = 1.36, 95% CI [1.20, 1.55]) compared to individuals between 45 and 64; this difference was not significant in 2021 (AOR = 1.32, 95% CI [0.99, 1.78]).

In 2021, there were conflicting variations in delayed care between regions. Hispanic and Asian individuals in the South had a higher odds of delaying care compared to White individuals (AOR_Hispanic_ = 1.17, 95% CI [1.03, 1.34]; AOR_Asian_ = 1.31, 95% CI [1.03, 1.66]); individuals in these demographic groups had a lower odds of delaying care in the West (AOR_Hispanic_ = 0.71, 95% CI [0.62, 0.80]; AOR_Asian_ = 0.77, 95% CI [0.64, 0.92]). In addition, individuals in the South without a high school degree had a higher odds of delaying care compared to college graduates (AOR = 1.24, 95% CI [1.01, 1.52] opposed to the Northeast (AOR = 0.70, 95% CI [0.52, 0.94]). Also in the Northeast, individuals without health insurance had higher odds of delaying care compared to those who had insurance from their employer (AOR = 1.43, 95% CI [1.06, 1.92]). Finally, individuals making above $100,000 in the Midwest had lower odds of delaying care compared to those making between $50,000 and $100,000 (AOR = 0.75, 95% CI [0.62, 0.91]; see Supplementary Materials 1 for a complete list of comparisons).

### 3.2 Underlying health conditions

A weighted 31.3% of respondents indicated “good” overall health. Fewer reported poor (2.34%) and fair (11.70%) health than very good (36.90%) and excellent (17.80%) health. Individuals who delayed medical care were more likely to have worse overall health when accounting for demographic covariates (Table 2). Compared to those who indicated good overall health, those reporting poor (AOR = 2.62, 95% CI [2.47, 2.78]) and fair (AOR = 1.68, 95% CI [1.61, 1.74]) health were associated with delaying care, adjusting for demographic covariates. Conversely, individuals who reported very good (AOR = 0.64, 95% CI [0.63, 0.66]) and excellent (AOR = 0.40, 95% CI [0.39, 0.41]) health were progressively less likely to delay care.

**Table 2:** Overall Health and Likelihood to Delay Medical Care.

	Overall (N = 312,661)^a^			2020 (N = 212,697)^a^			2021 (N = 99,964)^a^		
Overall Health	%^b^	AOR^c^	(95% CI)^d^	%^b^	AOR^c^	(95% CI)^d^	%^b^	AOR^c^	(95% CI)^d^
Poor	2.34	**2.62**	(2.47, 2.78)	2.02	**2.53**	(2.30, 2.80)	4.39	**4.14**	(3.61, 4.75)
Fair	11.70	**1.68**	(1.61, 1.74)	11.50	**1.70**	(1.63, 1.77)	13.40	**1.84**	(1.70, 1.99)
Good^e^	31.30	–	–	31.60	–	–	29.40	–	–
Very Good	26.90	**0.64**	(0.63, 0.66)	37.50	**0.61**	(0.60, 0.62)	32.60	**0.67**	(0.63, 0.71)
Excellent	17.80	**0.40**	(0.39, 0.41)	17.40	**0.38**	(0.37, 0.40)	20.10	**0.45**	(0.42, 0.49)

Boldface indicates statistical significance (p < 0.05).

a Unweighted N.

b Weighted proportion from total population.

c Adjusted Odds Ratio (AOR); adjusted for age, gender, race, annual household income, insurance status, and education status. Incorporated survey weights.

d Confidence interval (CI).

e “Good” health used as reference for AORs.

In 2020, there was a negative relationship between likelihood to delay care and overall health (Table 2). Those with poor (AOR = 2.53, 95% CI [2.30, 2.80]) health were more likely to delay care; those with excellent (AOR = 0.38, 95% CI [0.37 to 0.40]) health were less likely to delay care compared to those with good health. While delaying medical care was less prevalent in 2021, those with poorer health and underlying conditions were still more likely to delay medical care (Table 2). Individuals with poor (AOR = 4.14, 95% CI [3.61, 4.75]) health were even more likely to delay care in 2021. Those with excellent (AOR = 0.45, 95% CI [0.42, 0.49]) health had a similar association with delaying care in 2020.

Overall, 30.7% of respondents had at least one underlying condition. Individuals with underlying conditions had a higher likelihood of delaying medical care when accounting for demographic covariates. Those with any underlying conditions (AOR = 1.62, 95% CI [1.58, 1.65]) were associated with delaying care compared to those without a listed underlying condition. Each underlying condition observed an association with delaying care (Table 3). Delaying medical care was most likely among those with immunosuppressive conditions (AOR = 2.46, 95% CI [2.37, 2.58]) and chronic lung disease (AOR = 2.36, 95% CI [2.23, 2.51]). Individuals with chronic kidney disease (AOR = 2.16, 95% CI [2.04, 2.29]) and chronic heart disease (AOR = 2.10, 95% CI [2.02, 2.18]) were almost twice as likely to have delayed care compared to those without each condition (Table 3).

**Table 3:** Underlying Conditions and Likelihood to Delay Medical Care.

	Overall (N = 312,661)^a^			2020 (N = 212,697)^a^			2021 (N = 99,964)^a^		
Underlying Conditions	%^b^	AOR^c^	(95% CI)^d^	%^b^	AOR^c^	(95% CI)^d^	%^b^	AOR^c^	(95% CI)^d^
Any condition	30.70	**1.62**	(1.58, 1.65)	27.40	**1.80**	(1.77, 1.84)	37.80	**2.46**	(2.32, 2.61)
Asthma	17.30	**1.65**	(1.62, 1.68)	16.50	**1.63**	(1.57, 1.70)	22.60	**2.08**	(1.96, 2.20)
Cancer in the past year	2.68	**1.80**	(1.70, 1.91)	2.47	**1.62**	(1.49, 1.75)	4.05	**3.32**	(2.89, 3.81)
Chronic Heart Disease	5.44	**2.10**	(2.02, 2.18)	5.17	**1.97**	(1.86, 2.09)	7.24	**3.35**	(3.04, 3.70)
Chronic Kidney Disease	2.36	**2.16**	(2.04, 2.29)	1.99	**1.95**	(1.81, 2.11)	4.76	**4.48**	(3.98, 5.04)
Chronic Lung Disease	4.04	**2.36**	(2.23, 2.51)	3.59	**2.08**	(1.96, 2.20)	6.91	**4.81**	(4.27, 5.41)
Diabetes	11.50	**1.42**	(1.36, 1.48)	11.40	**1.46**	(1.41, 1.52)	12.30	**1.72**	(1.59, 1.86)
Immunosuppressive	6.97	**2.46**	(2.37, 2.58)	6.91	**2.48**	(2.39, 2.58)	7.34	**2.72**	(2.46, 3.00)

Boldface indicates statistical significance (p < 0.05).

a Unweighted N.

b Weighted proportion from total population.

c Adjusted Odds Ratio (AOR); adjusted for age, gender, race, annual household income, insurance status, and education status. Incorporated survey weights.

d Confidence interval (CI).

A higher proportion of respondents reported having at least one underlying condition in 2021 (37.80%) than 2020 (27.40%), excluding obesity (Table 3). In 2020, delayed care was more likely among individuals with any underlying condition (AOR = 1.80, 95% CI [1.77, 1.84]), particularly chronic heart disease (AOR = 1.97, 95% CI [1.86, 2.09]), chronic lung disease (AOR = 2.08, 95% CI [1.96, 2.20]), and immunosuppressive conditions (AOR = 2.48, 95% CI [2.39, 2.58]). Delaying medical care was also highly associated with chronic lung disease (AOR = 4.81, 95% CI [4.27, 5.41]) in 2021. Individuals with chronic kidney disease (AOR = 4.48, 95% CI [3.98, 5.04]), chronic heart disease (AOR = 3.35, 95% CI [3.04, 3.70]), and cancer in the past year (AOR = 3.32, 95% CI [2.89, 3.81]) were also likely to delay care. Obesity was only available in 2021 data but was also found to be associated with delaying medical care (AOR = 1.68, 95% CI [1.59, 1.78]).

### 3.3 Regional underlying health condition Analysis

**Fig. 1:**
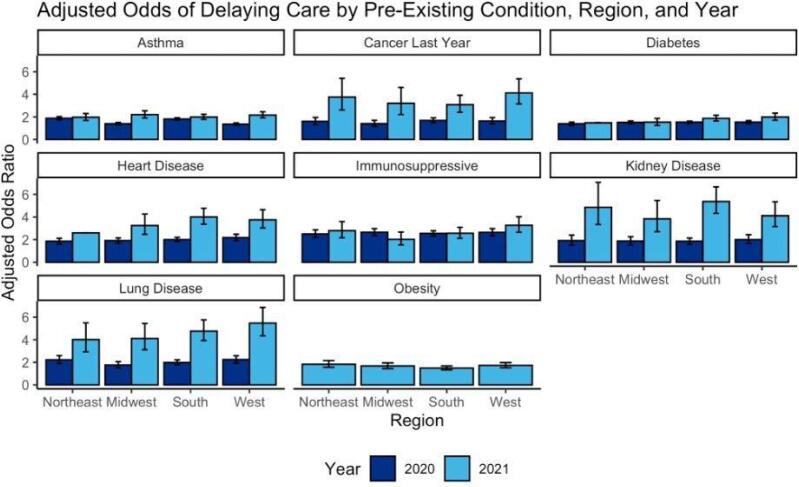
Comparison of adjusted odds (AOR) of delaying care across health condition, region, and year. AOR are derived from mixed-effects models with a random effect for state and stratified by Census region and year. All models are adjusted for age, gender, race, annual household income, insurance status, and education status.

Across underlying conditions, adjusted odds of delaying care generally increased between 2020 and 2021 by region (Fig. 1). The only exception was those with immunosuppressive diseases in the Midwest (AOR_2020_ = 2.65, 95% CI [2.36, 2.97] vs. AOR_2021_ = 2.02, 95% CI [1.54, 2.67]). In 2020, the Northeast and Midwest had the highest odds of delaying care for one condition each (asthma and immunosuppressive diseases, respectively), compared to two in the South (cancer and diabetes) and four in the West (diabetes, heart disease, kidney disease, and lung disease). In 2021, those with obesity or asthma had the highest odds of delaying care in the Northeast and Midwest respectively, compared to those with heart disease or kidney disease in the South or those with cancer, diabetes, immunosuppressive disease, or lung disease in the West (see Supplementary Materials 2 for complete comparisons).

## 4 Discussion

Understanding associations between delayed medical care, demographics, and individual health status are important to anticipate potential long-term health implications of the COVID-19 pandemic. To our knowledge, this work is the first to compare variations in delayed care from the early stages (April - June 2020) of the pandemic to later stages (April - June 2021) in the U.S. In 2020 and 2021, discrepancies in delayed medical care were present across health status, socioeconomic status, and geographic regions. The majority of those who delayed medical care in 2021 reported having at least one pre-existing condition (55.8%) which may be exacerbated after delaying care (Czeisler, 2020). Taken together, these findings demonstrate that the public health impact of delayed care is likely widespread and evolving.

Delayed medical care varied by socioeconomic status across years. More individuals with a household income of at least $100,000 reported delaying care in 2020, while a higher proportion of those with a lower income (below $50,000) reported delaying care in 2021. Those with a lower household income likely experienced a higher level of financial strain throughout the pandemic, which may have impacted their ability to receive medical care in 2021 (Parker et al., 2020, Horowitz et al., 2021). Prior work has demonstrated a direct relationship between socioeconomic status (SES) and COVID-19 outcomes, and the present work adds to this by elucidating a potential mechanism by which this occurs; that SES may lead to employment changes that adversely affect one’s ability to receive care. However, future work is needed to validate this mechanism.

In both 2020 and 2021, those with poorer overall health were more likely to delay medical care. As self-reported overall health improved, we observed a progressively lower likelihood to delay medical care. Similar to those with underlying conditions, there is concern surrounding exacerbation of poor health as a result of delayed medical care (Woolf et al., 2020, Lange, 2020, Chanchlani et al., 2022). It is important to consider that those with underlying conditions may generally seek more healthcare because of their conditions.

Furthermore, each underlying condition was associated with delaying medical care compared to the absence of the condition(s). Each of the assessed conditions could contribute to severe COVID-19 (CDC, 2020), so individuals with these conditions may have delayed care to mitigate potential COVID-19 exposure. Immunosuppressive conditions, chronic lung disease, chronic heart disease, and chronic kidney disease were especially associated with delaying medical care. Across conditions, there was an increased likelihood to delay care in 2021 than 2020, despite the overall population having a lower proportion of delayed care in 2021.

Regionally, there were higher rates of delayed medical care in the West and lower rates reported in the South. Interestingly, these variations do not parallel trends in case counts during the survey periods. Case counts were highest in the Northeast during the 2020 data collection period and in the Midwest in the 2021 collection period, yet the highest percentage of delayed care was in the West for both time periods (The New York Times, 2022). Additionally, among those that delayed care, COVID-19 rates did not impact their decision to delay. This may be explained in part by geographic variations in beliefs around the severity of the pandemic (The New York Times, 2022, Guntuku et al., 2021). However, an alternative reason could be that individuals consistently delay care for reasons that existed before COVID-19, such as a lack of access to health care facilities, health insurance, or paid sick leave.

A national survey from June 2020 found that individuals with pre-existing conditions had a high prevalence of delayed care, which aligns with the findings in this study from both 2020 and 2021 (Czeisler, 2020). That study also found that younger individuals (ages 18 to 24) had a higher odds of delaying care than older individuals (ages 25 to 44) (Czeisler, 2020). A similar finding was found within the present study. This finding is not unique to the pandemic era; studies conducted prior to COVID-19 also found that younger individuals delayed care largely due to financial reasons (Americans’ Views of Healthcare Costs, Coverage, and Policy, 2018). Given the rise of unemployment due to the pandemic, it is possible that economic hardship amplified by the pandemic magnified a barrier to care that was already highly problematic for younger persons (Kochhar, 2022).

There were some differences between our present study and the aforementioned study (Czeisler, 2020). While that study found that both Black and Hispanic individuals were more likely to delay care compared to White individuals, we found that Hispanic individuals in the West in 2020 and 2021 were less likely to delay care compared to White individuals. Hispanic individuals in the South had a lower odds of delaying care in 2020, and higher odds of delaying care in 2021. While the present study collected data between late-April and early-June of 2020, participants in the external study were polled in late June. The inter-region variations within each year demonstrated within the present study suggests the importance of evaluating the differential impacts between regions, especially due to variations in the impact of and response to COVID-19 between regions (Hallas et al., 2021).

Despite the strengths of the study, there are limitations. First, the study analyzes a limited time period which does not capture all stages of the pandemic, although this is partially addressed by including data from 2020 and 2021. The survey question on delaying medical care asked about the past four weeks, and there were no data on patients with obesity in 2020. We hypothesize that these individuals would be less likely to delay care during this period than in 2021, since the link between COVID-19 and obesity was not realized until after the initial polling period. Also, this study does not evaluate the type of care that was delayed, which has been studied for some diseases (COVID-19 significantly impacts health services for noncommunicable diseases, 2022, Woolf et al., 2020, Vervoort et al., 2020). Given the data were collected through a survey, results may be subject to recall bias. Furthermore, the survey uses a non probability–based sample and may not be generalizable to the U.S. population on unmeasured factors not incorporated into survey weights. Further study could seek to leverage clinical records or claims data to validate these findings or examine the relationship between lack of access to health services and the decision to delay care. Additionally, future work could also look at differences on a more granular geospatial level, such as state or county.

## 5 Conclusion

Delaying medical care can exacerbate existing medical conditions and may increase risk associated with preventable medical conditions (Czeisler, 2020, Masroor, 2020, CDC, 2022, Blay et al., 2021, Tapper and Asrani, 2020). Disparities among populations that delay medical care can have long-term implications on personal and public health. Findings presented here suggest that individuals with self-reported underlying conditions and poor health were predisposed to delay care throughout the pandemic. There were also distinct sociodemographic and regional differences between individuals and their likelihood of delaying care. Education on the importance of preventative and maintenance care should increase so individuals are aware of all potential impacts of delaying medical care. Indirect impacts from the pandemic should be considered when attributing population health effects to the pandemic, planning recovery efforts, and preparing for the next pandemic.

## CRediT authorship contribution statement

**Autumn H. Gertz:** Conceptualization, Methodology, Formal analysis, Data curation, Writing – original draft, Writing – review & editing, Project administration. **Catherine C. Pollack:** Methodology, Formal analysis, Writing – original draft, Writing – review & editing, Visualization. **Marinanicole D. Schultheiss:** Methodology, Writing – original draft, Writing – review & editing. **John S. Brownstein:** Writing – review & editing, Supervision, Project administration.

## Declaration of Competing Interest

The authors declare that they have no known competing financial interests or personal relationships that could have appeared to influence the work reported in this paper.
