# Supplementary material for: Delayed medical care and underlying health in the United States during the COVID-19 pandemic: A cross-sectional study
Source: Prev Med Rep. 2022 Jul 5;28:101882. doi: 10.1016/j.pmedr.2022.101882 (PMC9254505; doi:10.1016/j.pmedr.2022.101882)
Supplement: Supplementary Data 1 [file mmc1.docx]

| **Supplementary Materials 1. Regional Variations in Delayed Care by Sociodemographic Variables** | | | | | | | | |
| --- | --- | --- | --- | --- | --- | --- | --- | --- |
|  | 2020 (N = 212,697) | | | | 2021 (N = 99,964) | | | |
| Characteristic | Northeast  n = 38,347  AOR^a^ (95% CI^b^) | Midwest  n = 46,692  AOR (95% CI) | South  n = 74,846  AOR (95% CI) | West  n = 52,812  AOR (95% CI) | Northeast  n = 17,675  AOR (95% CI) | Midwest  n = 20,170  AOR (95% CI) | South  n = 37,012  AOR (95% CI) | West  n = 25,107  AOR (95% CI) |
| Pre-Existing Condition (Reference: No)  Yes | **1.91 (1.80, 2.02)** | **1.67 (1.58, 1.77)** | **1.93 (1.85, 2.01)** | **1.74 (1.65, 1.84)** | **2.08** **(1.82, 2.38)** | **2.59 (2.28, 2.94)** | **2.52 (2.30, 2.76)** | **2.87 (2.59, 3.18)** |
| Age (Reference: 45 – 64 years old)  13 – 17  18 – 24  25 – 44  65 and Over | 1.09 (0.95, 1.25)  **1.22 (1.11, 1.35)**  **1.08 (1.01, 1.17)**  0.94 (0.85, 1.03) | **1.36** **(1.20, 1.55)**  **1.10** **(1.01, 1.21)**  1.07 (1.00, 1.14)  0.99 (0.90, 1.09) | 1.05 (0.95, 1.16)  **1.16 (1.08, 1.25)**  **1.12** **(1.06, 1.18)**  0.99 (0.92, 1.06) | 0.91 (0.81, 1.02)  **1.13** **(1.04, 1.24)**  1.04 (0.98, 1.11)  **0.88 (0.80, 0.96)** | **1.86 (1.38, 2.50)**  **1.49 (1.19, 1.86)**  **1.46 (1.24, 1.73)**  **0.51 (0.39, 0.66)** | 1.32 (0.99, 1.78)  **1.99 (1.62, 2.45)**  **1.77 (1.51, 2.09)**  **0.46 (0.35, 0.60)** | **1.42 (1.16, 1.75)**  **1.75 (1.50, 2.05)**  **1.82 (1.61, 2.05)**  **0.56 (0.46, 0.68)** | **1.32 (1.04, 1.67)**  **1.70 (1.42, 2.03)**  **1.76 (1.54, 2.01)**  **0.63 (0.51, 0.76)** |
| Gender (Reference: Male)  Female  Other | **1.26 (1.19, 1.33)**  **1.50** **(1.18, 1.91)** | **1.46** **(1.39, 1.54)**  **2.15 (1.76, 2.61)** | **1.48 (1.42, 1.55)**  **2.35 (2.02, 2.73)** | **1.43** **(1.36, 1.50)**  **2.14** **(1.81, 2.53)** | **1.40 (1.22, 1.60)**  **4.11 (3.03, 5.58)** | **1.33 (1.17, 1.51)**  **2.40 (1.74, 3.30)** | 1.10 (1.00, 1.21)  **1.90 (1.49, 2.44)** | **1.14 (1.03, 1.26)**  **2.94 (2.33, 3.72)** |
| Race (Reference: White)  Black  Hispanic  Asian  Other | **0.77** **(0.70, 0.85)**  1.01 (0.92, 1.11)  0.93 (0.83, 1.05)  1.17 (0.98, 1.40) | **0.71 (0.64, 0.78)**  1.00 (0.90, 1.12)  **0.72 (0.62, 0.85)**  **1.21** **(1.01, 1.44)** | **0.71** **(0.66, 0.75)**  **0.81** **(0.76, 0.86)**  **0.65** **(0.58, 0.73)**  **1.14** **(1.02, 1.28)** | **0.86 (0.78, 0.94)**  **0.78** **(0.72, 0.83)**  **0.73 (0.67, 0.80)**  0.99 (0.85, 1.14) | 1.04 (0.83, 1.30)  1.14 (0.94, 1.38)  0.97 (0.75, 1.25)  **1.56** **(1.08, 2.26)** | 0.82 (0.67, 1.02)  1.13 (0.92, 1.40)  1.11 (0.81, 1.53)  1.18 (0.79, 1.77) | 0.95 (0.83, 1.07)  **1.17 (1.03, 1.34)**  **1.31 (1.03, 1.66)**  **1.33 (1.02, 1.74)** | 1.17 (0.94, 1.44)  **0.71 (0.62, 0.80)**  **0.77 (0.64, 0.92)**  0.79 (0.60, 1.03) |
| Income (Reference: 50,000 – 99,999 USD)  Below 50,000  100,000 and Above | **1.10** **(1.02, 1.18)**  **1.09** **(1.01, 1.17)** | **1.08** **(1.01, 1.15)**  **1.12** **(1.04, 1.20)** | 1.03 (0.98, 1.09)  1.05 (1.00, 1.11) | 1.06 (0.99, 1.13)  1.07 (1.00, 1.15) | 1.13 (0.95, 1.35)  0.95 (0.79, 1.14) | **1.21 (1.03, 1.41)**  **0.75 (0.62, 0.91)** | **1.40 (1.24, 1.57)**  1.10 (0.96, 1.27) | **1.20 (1.06, 1.37)**  0.97 (0.84, 1.12) |
| Insurance Source (Reference: Employer)  Purchased oneself  Medicare  Medicaid or Medi-Cal  TRICARE  None  Other  No Answer | 0.91 (0.82, 1.00)  0.99 (0.91, 1.08)  **1.20 (1.07, 1.34)**  1.26 (0.59, 1.66)  0.97 (0.85, 1.12)  **0.79 (0.70, 0.90)**  0.87 (0.59, 1.29) | **0.89** **(0.81, 0.98)**  1.06 (0.97, 1.15)  **1.16** **(1.04, 1.30)**  1.09 (0.85, 1.40)  **0.81** **(0.72, 0.92)**  **0.82 (0.73, 0.93)**  0.68 (0.40, 1.14) | 0.93 (0.87, 1.00)  **1.08 (1.01, 1.16)**  **1.24 (1.14, 1.35)**  1.14 (1.00, 1.29)  **0.83** **(0.77, 0.89)**  0.93 (0.85, 1.00)  0.82 (0.60, 1.12) | **0.88 (0.81, 0.96)**  0.98 (0.90, 1.06)  1.03 (0.94, 1.12)  1.15 (0.98, 1.36)  **0.81** **(0.72, 0.90)**  **0.84** **(0.76, 0.94)**  **0.59** **(0.39, 0.91)** | 1.02 (0.79, 1.31)  **1.27** **(1.04, 1.56)**  **1.84** **(1.46, 2.31)**  1.37 (0.76, 2.47)  **1.43 (1.06, 1.92)**  0.97 (0.72, 1.30)  1.87 (1.00, 3.49) | **1.30 (1.05, 1.62)**  **1.43 (1.17, 1.74)**  **1.43 (1.15, 1.78)**  1.31 (0.81, 2.11)  1.09 (0.84, 1.41)  1.21 (0.92, 1.61)  0.65 (0.25, 1.70) | 1.13 (0.97, 1.32)  **1.31 (1.13, 1.53)**  **1.29 (1.10, 1.52)**  1.13 (0.85, 1.50)  1.03 (0.88, 1.21)  0.82 (0.66, 1.01)  0.69 (0.39, 1.22) | 1.09 (0.92, 1.29)  **1.39 (1.18, 1.63)**  1.00 (0.84, 1.18)  0.76 (0.51, 1.13)  0.96 (0.77, 1.19)  0.93 (0.75, 1.15)  1.64 (0.97, 2.79) |
| Education (Reference: College Degree)  Did not finish high school  High School/GED  Associate’s Degree  Some College  Postgraduate Degree | **0.74** **(0.64, 0.84)**  **0.63** **(0.58, 0.69)**  **0.84** **(0.75, 0.94)**  **0.89** **(0.81, 0.98)**  1.12 (0.99, 1.28) | **0.57** **(0.50, 0.66)**  **0.62 (0.57, 0.67)**  0.90 (0.81, 1.00)  0.92 (0.84, 1.00)  1.01 (0.86, 1.17) | **0.67** **(0.60, 0.73)**  **0.59 (0.56, 0.63)**  **0.81** **(0.75, 0.88)**  **0.86 (0.81, 0.93)**  **1.14 (1.03, 1.26)** | **0.69** **(0.62, 0.78)**  **0.67** (**0.63, 0.73)**  **0.86** **(0.78, 0.95)**  **0.82** **(0.76, 0.89)**  1.07 (0.94, 1.22) | **0.70 (0.52, 0.94)**  **0.69 (0.56, 0.84)**  1.01 (0.77, 1.33)  0.83 (0.67, 1.04)  **1.37 (1.08, 1.73)** | 1.09 (0.83, 1.45)  **0.77 (0.63, 0.94)**  0.78 (0.60, 1.02)  **0.78 (0.63, 0.96)**  1.26 (0.95, 1.66) | **1.24 (1.01, 1.52)**  **0.77 (0.67, 0.90)**  1.04 (0.86, 1.26)  0.99 (0.84, 1.16)  **1.27 (1.03, 1.57)** | 0.95 (0.76, 1.18)  **0.81 (0.69, 0.95)**  1.02 (0.83, 1.24)  **0.84 (0.71, 0.99)**  1.15 (0.92, 1.43) |
| ^a^ AOR: Adjusted Odds Ratio  ^b^ CI: Confidence Interval  All models are weighted mixed-effects logistic regression with a random intercept for state, adjusted for age, sex, race, income, insurance, and education. Values above 1 denote greater odds of delaying care, while values below 1 denote lower odds of delaying care.  Boldface indicates statistical significance (p<0.05). | | | | | | | | |

| **Supplementary Materials 2. Regional Variations in Delayed Care by Pre-Existing Condition** | | | | | | | | |
| --- | --- | --- | --- | --- | --- | --- | --- | --- |
| Parameter | Northeast | | Midwest | | South | | West | |
|  | 2020 | 2021 | 2020 | 2021 | 2020 | 2021 | 2020 | 2021 |
|  | AOR^a^ (95% CI^b^) | AOR (95% CI) | AOR (95% CI) | AOR (95% CI) | AOR (95% CI) | AOR (95% CI) | AOR (95% CI) | AOR (95% CI) |
| Asthma | **1.88 (1.75, 2.02)** | **1.97 (1.68, 2.30)** | **1.39 (1.20, 1.50)** | **2.20 (1.90, 2.54)** | **1.81 (1.72, 1.92)** | **1.99 (1.78, 2.23)** | **1.36 (1.27, 1.46)** | **2.16 (1.92, 2.45)** |
| Cancer | **1.60 (1.23, 1.95)** | **3.76 (2.61, 5.42)** | **1.40 (1.16, 1.69)** | **3.20 (2.21, 4.61)** | **1.68 (1.48, 1.92)** | **3.08 (2.41, 3.92)** | **1.63 (1.37, 1.94)** | **4.12 (3.15, 5.38)** |
| Diabetes | **1.39 (1.26, 1.53)** | **1.47 (1.47, 1.48)** | **1.51 (1.39, 1.64)** | **1.53 (1.25, 1.87)** | **1.53 (1.43, 1.62)** | **1.87 (1.63, 2.15)** | **1.53 (1.41, 1.67)** | **1.99 (1.70, 2.33)** |
| Heart Disease | **1.85 (1.61, 2.12)** | **2.59 (2.58, 2.60)** | **1.91 (1.69, 2.15)** | **3.25 (2.47, 4.26)** | **2.00 (1.82, 2.20)** | **4.01 (3.37, 4.78)** | **2.18 (1.91, 2.48)** | **3.75 (3.03, 4.65)** |
| Immunosuppressive | **2.50 (2.19, 2.86)** | **2.79 (2.17, 3.59)** | **2.65 (2.36, 2.97)** | **2.02 (1.54, 2.67)** | **2.55 (2.33, 2.78)** | **2.56 (2.12, 3.09)** | **2.64 (2.35, 2.97)** | **3.27 (2.65, 4.03)** |
| Kidney Disease | **1.91 (1.52, 2.40)** | **4.86 (3.34, 7.07)** | **1.86 (1.53, 2.26)** | **3.84 (2.70, 5.46)** | **1.85 (1.60, 2.14)** | **5.37 (4.32, 6.67)** | **2.00 (1.65, 2.43)** | **4.11 (3.16, 5.35)** |
| Lung Disease | **2.22 (1.90, 2.60)** | **4.01 (2.93, 5.49)** | **1.76 (1.51, 2.06)** | **4.11 (3.11, 5.44)** | **1.98 (1.78, 2.22)** | **4.76 (3.93, 5.76)** | **2.24 (1.93, 2.59)** | **5.47 (4.35, 6.86)** |
| Obesity | -^c^ | **1.83 (1.56, 2.15)** | - | **1.68 (1.44, 1.95)** | - | **1.49 (1.33, 1.68)** | - | **1.73 (1.52, 1.97)** |
| ^a^ AOR: Adjusted Odds Ratio  ^b^ CI: Confidence Interval  ^c^ Obesity was not listed as a pre-existing condition in the 2020 surveys  All models are weighted mixed-effects logistic regression with a random intercept for state, adjusted for age, sex, race, income, insurance, and education. Values above 1 denote greater odds of delaying care.  Boldface indicates statistical significance (p<0.05). | | | | | | | | |
